# Real-Time Observation of Carbon Oxidation by Driven Motion of Catalytic Ceria Nanoparticles within Low Pressure Oxygen

**DOI:** 10.1038/s41598-019-44531-6

**Published:** 2019-05-30

**Authors:** Boyu Li, Anton D. Sediako, Pei Zhao, Jingde Li, Eric Croiset, Murray J. Thomson, John Z. Wen

**Affiliations:** 1 Department of Chemical Engineering, University of Waterloo, 200 University Avenue West, Waterloo, ON N2L 3G1 Canada; 2 Department of Mechanical & Industrial Engineering, University of Toronto, 5 King’s College Road, Toronto, ON M5S 3G8 Canada; 3 Department of Mechanical & Mechatronics Engineering, University of Waterloo, 200 University Avenue West, Waterloo, ON N2L 3G1 Canada

**Keywords:** Environmental sciences, Fossil fuels

## Abstract

Carbon particulate matter (PM) is an undesirable aerosol pollutant formed from combustors such as power plants, refineries, and engines. The most common and effective method of mitigating PM emission is the capture of particulates using a filter, before particles are released into the atmosphere. In order to develop and improve advanced filtering materials, a better understanding is required of their chemical and mechanical behavior. We report on a novel phenomenon on the mobility and oxidation behavior of catalytic iron doped ceria nanoparticles in contact with mobile carbon black nanoparticles. The process is recorded by real time imaging within an environmental transmission electron microscope. In contrast to observations in previous studies, the separated ceria nanoparticles are found to actively move on the substrate and consume the connecting carbon particles one-by-one. The velocity of particle motion is correlated to the reaction temperature and oxygen pressure, both determining the reaction rate. Modeling using the Density Functional Theory suggests this motion is driven by the chemical bonding between the surface oxygen of the catalyst and the graphite layers of carbon black, initiated through the Van der Waals force between two types of nanoparticles.

## Introduction

There is an urgent need to develop novel materials for capturing and reducing particulate emissions from a wide range of combustion sources. Soot, as one type of particulate matter (PM), is an undesirable aerosol pollutant which forms in fossil fuel power plants, refineries, and internal combustion engines^1,2^. These fine carbonaceous particles, when released into the atmosphere, have been shown to be carcinogenic and directly linked to an increase in mortality rates^3^. For many sources, the most common and effective method of mitigating carbon PM emission is the capture of particulates using a filter, before they enter into the atmosphere. Advanced materials have been sought for developing high-efficiency filters, especially for these particulates with a size below 10 nm. For internal combustion engines, a diesel particulate filter (DPF) is usually installed in line with the exhaust stream from the engine^4^. When the DPF is saturated with the deposited particulates, it must be cleaned or replaced, to avoid increasing the back-pressure of the engine which affects the vehicle operation^5^. In order to reduce the cost of adding extra fuel to burn out the deposited particulates, a catalyst can effectively reduce the oxidation temperatures of carbon PM to 200–400 °C which is close to the temperature of engine exhaust gases^6^. DPFs utilizing the catalysts can be categorized by the method through which the catalyst is introduced: DPF enhanced with a catalyst coating directly on its wall (DPF-CC) and DPF utilizing fuel borne catalysts (DPF-FBC). For DPF-CC, a small amount of catalysts is immobilized as a thin coating (20 μm^7^) on the wall of DPF, which can reduce the oxidation temperature during regeneration. In a DPF-FBC, the catalyst is added directly to the fuel at a concentration of around 10ppm and reaches the DPF to oxidize carbon PM at a lower temperature^8–10^. For both type of enhanced DPF, the contact points between the carbon and catalysts is a critical factor for determining the process efficiency and the cost of the device, as catalytic carbon PM oxidation is a surface sensitive reaction. In literature, the structures of the coated catalyst and carbon deposition were found to affect the capability of a DPF-CC^11^. It was suggested that a porous catalyst coating leads to more contact points between catalyst and carbon PM^7^. For DPF-FBC, a homogeneous dispersion as well as intimate contact between catalyst and carbon PM contributes to better DPF performance^8,9^.

Ceria (CeO_2_) has been shown to be a good candidate for DPF-CC catalyst, as it is naturally abundant, less sensitive to sulfur impurities which poison the catalytic oxidation of carbon PM, and less environmentally toxic during end-of-life recycling^12–19^. Recently, in order to improve the activity and stability of ceria catalyst, metal cations such as Fe and Mn have been doped into the ceria lattice. These dopants effectively inhibit catalyst sintering and increase active sites, both improving the catalyst durability and activity^16,20–23^. The oxidation mechanisms of carbon particles using a pure CeO_2_ and a Fe/CeO_2_ catalyst have been investigated by Liu *et al*^24,25^. For the reaction with pure CeO_2_, ceria provides oxygen for carbon oxidation, followed by refilling of environmental oxygen into its crystal accompanied by the redox cycle between Ce^4+^ and Ce^3+^. As for carbon oxidation with the Fe/CeO_2_ catalyst, the redox cycle between Fe^2+^ and Fe^3+^ provides oxygen to oxidize carbon, and the redox of Ce continuously provides oxygen to re-oxidize Fe^2+^. Although many methods exist to dope metal ions into the ceria catalyst, recently it was found that 10% iron doping into a spongy ceria catalyst, prepared through combustion synthesis, produces the best results in terms of carbon oxidation rate and reduced oxidation temperature^26^.

During the catalyzed oxidation of carbon particles by the ceria catalyst, the mobility of these particles were found to affect the reaction rate^14,27^. Environmental transmission electron microscopy (ETEM) is a promising technology for revealing the interaction between nano-sized carbon and catalyst particles during reaction, by allowing the observation of particle shape changes and motions in a controlled temperature and gas pressure atmosphere^13,28–31^. Sediako *et al*. used ETEM to observe mature and less mature soot oxidation in real time. It was found that mature soot was oxidized on its surface, while the less mature soot showed internal oxidation^28^. Kamatani *et al*. utilized *in situ* Transmission Electron Microscopy (TEM) to visualize the catalytic soot oxidation using Ag/SiO_2_ and Cs_2_CO_3_ under 300 °C with 0.5 Pa O_2_^29^. They observed the catalytic reaction happened at the soot-catalyst interface with mobile Ag species and immobile Cs based catalyst. The mobility of Ag/SiO_2_ catalyst is considered as a result of heat released from carbon oxidation and partial liquefaction of Ag surface, while the fixed Cs_2_CO_3_ was due to strong interaction with the substrate. Gao *et al*. applied ETEM to investigate Ag catalyst on alumina and sulphated alumina supports for soot oxidation. Sulphated alumina was able to anchor the Ag catalyst thus making carbon particles move towards Ag species^32^. Baker *et al*. used a controlled atmosphere electron microscope (CAEM) to investigate the catalyst (2–5 nm) behavior during the gasification of carbon and graphite particles (original in 1 *μ*m, 2–20 nm after treatment)^33,34^. They proposed the concept of channeling catalysis, during which the reaction begins at the catalyst/graphite interface and proceeds through channels generated within the graphite crystal. Simonsen *et al*. studied the oxidation of carbon black (30 nm) using CeO_2_ catalyst agglomerate (50–100 nm) and observed that these carbon particles moved to reach CeO_2_, followed by the catalytic combustion at the carbon/catalyst interface^14^. Another study by Mori *et al*. reported the similar motion of carbon black particles (around 10–20 nm) towards an aggregated catalyst cluster made of Ag/CeO_2_ and Cu/BaO/La_2_O_3_ cluster (around 200–300 nm)^5^. Others reported the motion of carbon particles of around 20 nm that were approaching an agglomerate of yttria-stabilized zirconia catalysts during the catalytic oxidation^35^. Interestingly there has been no report in the literature on observing the nano-sized ceria based catalyst moving towards the carbon particles, which could provide insight on particle-particle interaction and subsequently the reaction mechanism of catalyzed oxidation of particulate matters.

In this work, the research focus is on the visualization and analysis of relative movement and interaction between carbon black nanoparticles and Fe/CeO_2_ catalyst nanoparticles, using the commercial CeO_2_ catalyst as a reference. We first report the movement of Fe/CeO_2_ catalyst nanoparticles towards carbon nanoparticles under the extremely low pressure of oxygen (<1 Pa) within an ETEM. The motion of the nanoparticles is analyzed using the catalyst activity and Density Functional Theory (DFT) modeling.

## Methodology

The ETEM experiments were conducted by using a Hitachi HF3300 ETEM equipped with Energy-dispersive X-ray spectroscopy (EDX), gas injection and sample heating. *In-situ* observation videos with spatial resolution of 0.2 nm can be recorded under desired temperature and gas atmosphere, particularly 500 °C or 800 °C, and 1 Pa or 0.2 Pa O_2_. Time-lapsed ETEM videos were obtained *in situ* of carbon nanoparticles in contact with catalyst as well as control experiments with only carbon nanoparticles.

Printex-U carbon black powder with an average particle size of 25 nm was used as a model of carbon nanoparticles, which is common for research on carbon PM oxidation^25,35–37^. The catalyst used was a 10% iron doped ceria catalyst (10%Fe/CeO_2_) with a size around 20 nanometers after grinding for 10 minutes, synthesized by means of the solution combustion synthesis method^38^. The crystal structure and morphology of the catalyst were investigated by German Bruker D4 (40 kV, 30 mA, CuKα radiation) X-ray Diffraction (XRD) and Scanning Electron Microscopy (SEM, Zeiss MERLIN with Gemini-II column). Carbon and catalyst were mixed by grinding in a weight ratio of 1:9^39^. The mixtures were then drop coated onto microelectrical mechanical system (MEMS) substrates (Norcada Inc. Model: HTN-010). The distribution of the catalyst and carbon particles on the substrate before and after the reaction was measured by EDX elemental mapping.

To enhance the contrast and increase the resolution, all imaging was done at 300 kV. Sensitivity tests were also carried out to ensure that the observed particle movement were not induced by the electron beam, the gas flow, or the sample/substrate interactions (^28^ and Supplementary Information). All tests were conducted according to the procedures established by a previous study as follows^28^: (1) Adjusted beam with sample within high vacuum; (2) Sample pre-heated to 400 °C and then inserted into the beam path. (3) Particles selected, focused and recorded; (4) Temperature raised to experiment temperature at 5 °C/s; (5) O_2_ or dry air injection started; (6) Conditions maintained until the reaction completes; (7) Gas off and cool down.

For computational method, the DFT calculations were carried out using the VASP package^40,41^. The Perdew-Burke-Ernzerhof (PBE) functional was used for the exchange and correlation energy terms. The CeO_2_ and carbon were modeled using a Ce_3_FeO_8_ cluster and a two-layer 6 × 6 graphene supercell, respectively. To be more specific, three graphene supercells with no carbon defects, six carbon defects (6-C) and eighteen carbon (18-C) defects were considered to model the realistic chemical states of the carbon materials during the reaction. The calculations performed on this study were spin-unrestricted, and all the atoms were set free to relax. The vacuum height is set to 15 Å. The planewave cutoff was set to 400 eV. The k-space was sampled using a 2 × 2 × 1 Monkhorst–Pack grid. Structures are fully relaxed until the forces acting on the atoms are smaller than 0.03 eV/Å. Based on the fully relaxed structures, the cohesive energies (*E*_*coh*_) of Ce_3_FeO_8_ cluster on the graphene (G) supercells were calculated by the following equation:

1 $${E}_{coh}={E}_{{{\rm{Ce}}}_{3}{{\rm{FeO}}}_{8}-G}-{E}_{{{\rm{Ce}}}_{3}{{\rm{FeO}}}_{8}}-{E}_{G}$$

where ${E}_{{{\rm{Ce}}}_{3}{{\rm{FeO}}}_{8}-G}$ represents the total energy of the Ce_3_FeO_8_ cluster to the graphene structure, ${E}_{{{\rm{Ce}}}_{3}{{\rm{FeO}}}_{8}}$ is the energy of an isolated Ce_3_FeO_8_ cluster, *E*_*G*_ is the energy of the optimized graphene model before bonding to Ce_3_FeO_8_. A more negative *E*_*coh*_ corresponds to a stronger bonding system.

## Results and Discussions

### Characterization of catalyst and carbon nanoparticles

**Figure 1:**
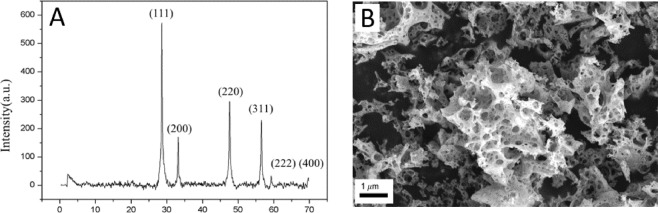
XRD pattern and SEM image of as-produced 10%Fe/CeO_2_ catalyst.

The XRD pattern of the 10%Fe/CeO_2_ catalyst is depicted in Fig. 1(A). The diffraction lines match the pattern of pure CeO_2_ with a cubic fluorite structure (Fm3m, JCPDS 34–0394), while no peak of any Fe_2_O_3_ crystal may be found, suggesting that iron incorporation into the CeO_2_ crystal lattice does not change the hosting crystalline structure. An SEM image of the catalyst is shown in Fig. 1(B). It shows the 10%Fe/CeO_2_ catalyst has a spongy/porous structure, which may enhance the contact to carbon nanoparticles and can facilitate diffusion of oxygen species to the carbon/catalyst interface.

**Figure 2:**
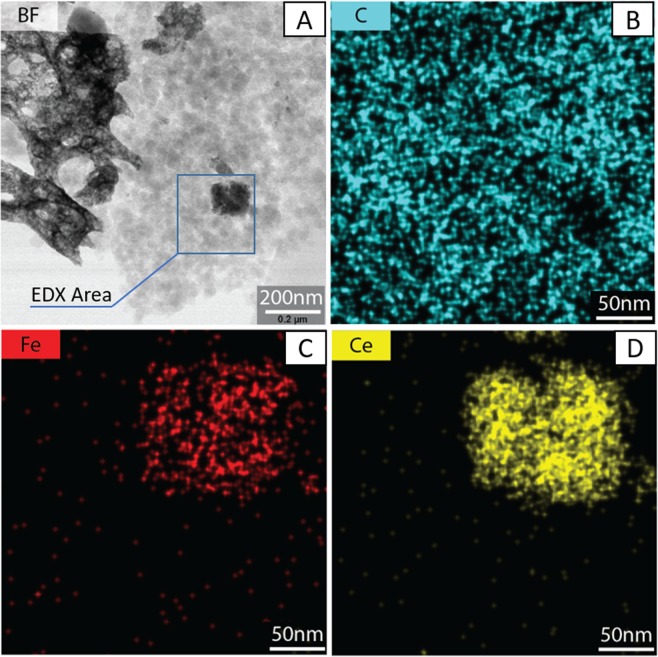
(**A**) TEM image of 10%Fe/CeO_2_ catalyst and carbon mixture, (**B**) EDX image for C, (**C**) EDX image for Fe, (**D**) EDX image for Ce.

TEM and EDX elemental mapping results of three elements (C, Fe, Ce) on the as-produced carbon-catalyst mixture are displayed in Fig. 2. Figure 2(A) is a bright field (BF) TEM image. Figure 2(B–D) are higher resolution EDX elemental maps for C, Fe and Ce, respectively, around a catalyst particle. In Fig. 2(A), the black feature represents catalyst particles, while the light grey feature represents carbon particles. This figure shows catalyst particles of different sizes, from large ones of over several hundred nanometers, to some about 100 nm, to very small ones under 10 nm. Figure 2(C,D) shows a zoom around a 100 nm catalyst particle, indicating that Fe is well dispersed on the Ceria support. The observed individual small dots for both Fe and Ce are caused by unavoidable noise due to the high resolution nature of the measurement.

### Mobility and Catalytic Oxidation in 1 Pa pure O_2_ at 500 °C

**Figure 3:**
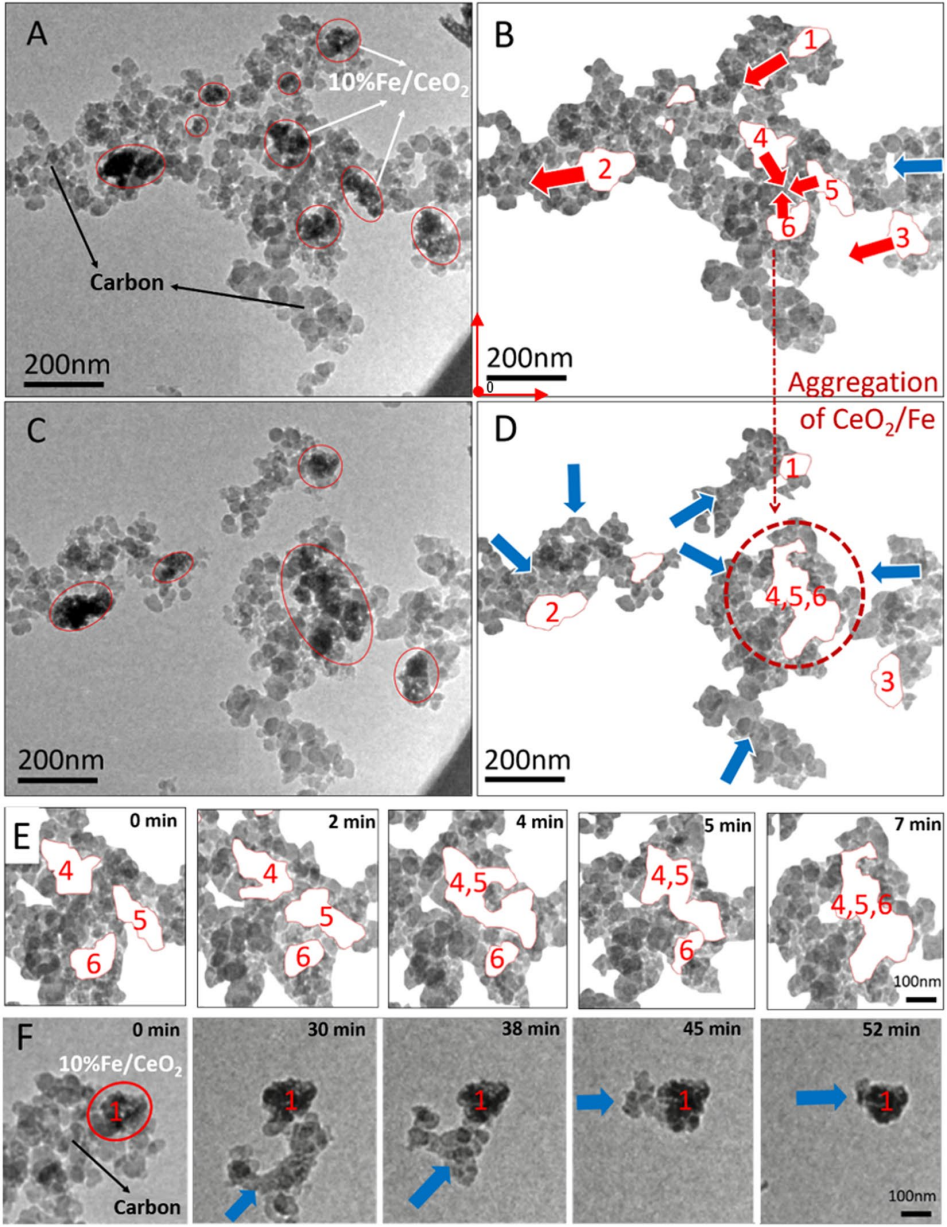
ETEM images of carbon and 10%Fe/CeO_2_ recorded at 500 °C in 1 Pa O_2_; (**A,B**) – 0 min; (**C,D**) – 7 min; (**B,D**) are processed images of (**A,C**), respectively, by cleaning the background, tracing 10%Fe/CeO_2_ clusters with a red outline and erasing 10%Fe/CeO_2_ to increase the contrast. The movement of carbon and 10%Fe/CeO_2_ is marked by blue and red arrows, respectively. (**E**) Movement and morphology change of catalysts marked ‘4, 5, and 6’ in (**B**). (**F**) Carbon moved towards the fixed catalyst marked ‘1’ in (**B**).

As shown in Supplementary Video and Fig. 3, when the catalyst was aggregated into a relatively small size dispersed among carbon, both the catalyst and carbon showed obvious movement during the oxidation in 1 Pa O_2_ at 500 °C, especially the small clusters of catalysts. Previous studies^13,14^ have shown the movement of carbon towards fixed ceria catalyst. However, this work provides the observation of the movement and restructuring of the 10%Fe/CeO_2_ catalyst.

**Figure 4:**
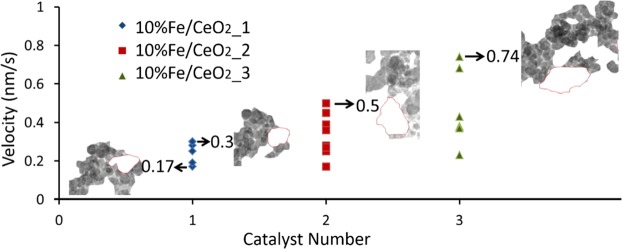
The velocity of 10%Fe/CeO_2_ clusters labeled as number 1, 2 and 3 in Fig. 3(B,D). For four values, the configuration of carbon and 10%Fe/CeO_2_ nanoparticles is given.

In Figs 3(B) and 4(D), the processed image of Figs 3(A) and 4(C), the movement of carbon was indicated by blue arrows and red arrows represented the catalyst movement. During the reaction, the catalysts oxidized the immediate surrounding carbon, and then began to actively travel and consume carbon along the trail of carbon. The interface and morphology of the catalyst cluster was always changing due to the varying configuration of the catalysts and their surrounding carbon, as shown in Fig. 3 (E). In the meantime, several adjacent catalyst clusters numbered as ‘4, 5, and 6’ in Fig. 3(B) tended to agglomerate into one cluster. As the carbon oxidation continued, the sizes/mass of carbon aggregates decreased to be relatively small compared with the catalysts. In this case, the catalyst cluster would stop moving and the surrounding carbon would keep moving towards it to maintain the reaction interface as shown in Fig. 3(F), which confirmed that the catalytic carbon oxidation occurred directly at the interface. One special observation was that the catalyst cluster labeled as number ‘3’ in Fig. 3(B) moved left together with surrounding carbon aggregation. Catalyst ‘3’ was pulled to left by connected mobile carbon, possibly driven by the cluster of catalyst circled in red in Fig. 3(D).

The analysis of the movement of the catalyst and carbon particles was carried out as follows: first the video was divided into several images at different times; at 500 °C, images were analyzed every minutes, whereas at 800 °C, images were analyzed every 30 seconds due to faster reaction. Each image was pixelated using ImageJ software who could assign coordinates to all pixels using a common reference origin (red point) as shown in Fig. 3(B). For Fig. 3, the pixel size is 0.83 nm by 0.83 nm. Once the boundary of the particles was determined (manually using Photoshop), the coordinate of its centroid was calculated using ImageJ. The movement velocity of the catalyst clusters labeled as number 1, 2 and 3 in Fig. 3(B) was tracked through the coordinate of its center, and the results are shown in Fig. 4. The velocity was obtained via dividing the displacement distance by the time interval of 1 min for a continuous period of 7 min and the movement velocity ranged from 0.17 to 0.74 nm/s. From Fig. 4, it was noticed that the mobility of catalyst was related to the configuration of the catalyst cluster and its surrounding carbon. The varying morphology of the catalyst cluster during carbon oxidation could change its center, and thereby its velocity, obtained by tracing the cluster center, involved not only the movement of the cluster towards carbon but also the shift of the cluster center. Apart from this, the velocity of catalyst particles was also influenced by the mobile carbon. For example, the catalyst cluster ‘3’ moved left against carbon, and its velocity was actually that of the surrounding carbon being pulled left by another larger catalyst cluster. Therefore, when the catalysts are dispersed in carbon in a complex configuration, it is reasonable to expect that their velocities will vary over a broad range.

**Figure 5:**
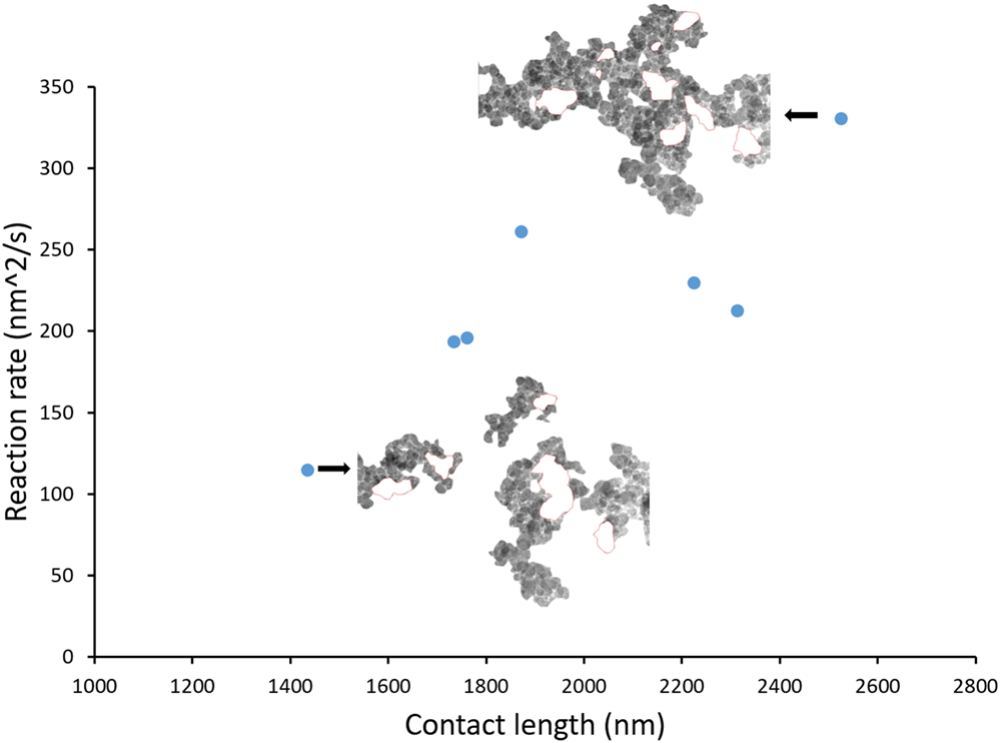
Correlation of contact length and reaction rate ‘r’ at 500 °C in 1 Pa O_2_. Two images show the corresponding contact lengths measured for two individual points in the figure.

The carbon oxidation rate (r) was estimated by measuring the decrease of the area (A) representing carbon particles at a given time. The methodology to determine the area (A) is described next. As mentioned previously, the contour of the catalyst particles was drawn manually using Photoshop. In a ETEM micrograph (e.g. Fig. 3), the black particles usually represent the catalyst, but not always, as several carbon particles existing on top of each other and would look dark grey or black. In order to avoid misrepresenting carbon particles as catalyst particles, those dark particles were monitored closely and carefully (if they shrunk or disappeared then it meant that they are carbon particles, otherwise they are considered as catalyst particles. Once all catalyst particles have been identified, they are artificially removed from the ETEM micrograph (e.g. see Fig. 3(B,D)). The area (A) considered for calculating the reaction rate was the entire surface area left on the ETEM micrograph (i.e. surface area representing carbon particles). The value of the area, A, was calculated using ImageJ. For each time interval, an oxidation rate along with a value of the catalyst velocity were obtained. As the catalytic reaction occurred at the interface and the reaction rate depended on the contact points between the carbon and catalyst, the reaction rates can be normalized (r′) by being divided by the contact length, as shown in Eqs (2) and (3). Being related to the movement of carbon and catalyst particles, the contact length varied from 1435 nm to 2530 nm and the reaction rate was measured in the range of 115–331 nm^2^/s. Figure 5 shows the relationship between the contact length and reaction rate ‘r’ at 500 °C in 1 Pa O_2_. This figure indicates that the reaction rate increases as the contact length increases.

2 $${\rm{Reaction}}\,{\rm{rate}}\,({\rm{based}}\,{\rm{on}}\,{\rm{area}}\,{\rm{decrease}}\,{\rm{in}}\,{\rm{a}}\,{\rm{certain}}\,{\rm{time}}\,{\rm{interval}})={\rm{r}}[\frac{{{\rm{nm}}}^{2}}{{\rm{s}}}]=\frac{dA\,[n{m}^{2}]}{dt[s]}$$

3 $${\rm{Normalized}}\,{\rm{reaction}}\,{\rm{rate}}({\rm{per}}\,{\rm{contact}}\,{\rm{length}})={\rm{r}}^{\prime} [\frac{{\rm{nm}}}{{\rm{s}}}]=\frac{r\,[\frac{n{m}^{2}}{s}]}{{\rm{contact}}\,{\rm{length}}\,[nm]}$$

In order to study the reaction kinetics, the data presented in Fig. 5 were used to calculate the normalized oxidation rate of carbon. Note that these data points in Fig. 5 were taken at different reaction times and therefore they are time dependent. Figure S2 in supplementary information gives the time dependent normalized reaction rate. For carbon oxidation at 500 °C (in both O_2_ and air), the normalized reaction rates were calculated to be a nearly constant value in the first 500 seconds. The normalized oxidation rate in 1 Pa pure O_2_ was hence determined as its average values of 0.11 ± 0.02 nm/s (the uncertainty was given as one standard deviation). The catalyst movement velocity was of the same order as the normalized oxidation rate at the interface, which implied that the mobility or the speed is correlated with the normalized reaction rate.

### Mobility of Catalyst Nanoparticles in 1 Pa O_2_ at 800 °C

**Figure 6:**
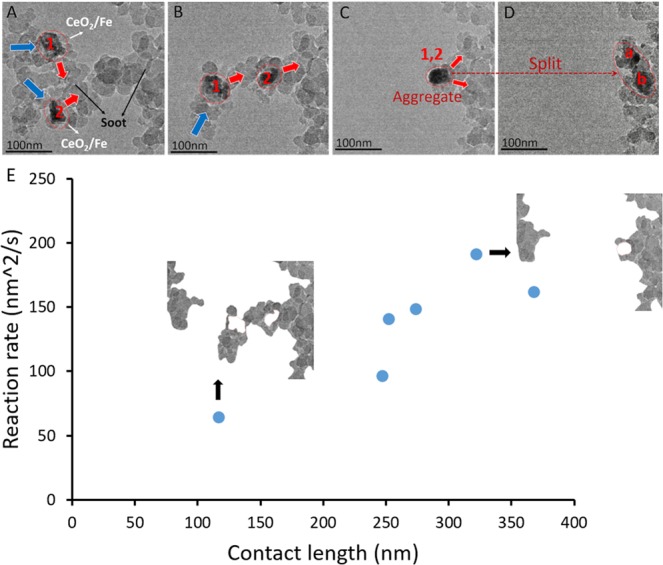
ETEM images of carbon and 10%Fe/CeO_2_ during the reaction at 800 °C in 1 Pa O_2_; (**A**) 0 min; (**B**) 2 min; (**C**) 4 min; (**D**) 6 min. The 10%Fe/CeO_2_ catalyst clusters are circled in red lines. (**E**) Correlation of contact length and reaction rate ‘r’.

To justify the movement of the catalyst and better correlate the movement of the catalysts to the reaction rate, the same experimental procedure was conducted at 800 °C in 1 Pa oxygen, as shown in Fig. 6. The increase in temperature caused the movement of catalyst, as shown by the red arrows, to become more rapid and active. Apart from the same observations in scenario of 1 Pa O_2_ at 500 °C, such as the movement of the catalysts and carbon, one notable phenomenon is that the catalyst aggregated as seen in Fig. 6(C) then separated as in Fig. 6(D), which is probably because they were pulled by carbon located at their upper right and lower right. It suggests that the catalyst clusters tend to aggregate instead of sintering as the reaction processes, because the sintered catalysts would not split afterwards.

With the movement of the catalysts and carbon, the contact length varied from 116 to 367 nm and the reaction rate was from 64 to 275 nm^2^/s. Figure 6(E) also shows that reaction rate has a positive relation with contact length. As shown in Figure S2 in supplementary information, the normalized reaction rate at 800 °C in O_2_ was calculated for the first 250 seconds of the reactions, due to fast oxidation of carbon particles. Those data were observed to be more fluctuating than these at 500 °C, because the particles moved more rapidly and along more irregular paths at a higher temperature. Nevertheless the average value of the normalized reaction rates in 1 Pa O_2_ at 800 °C was calculated as 0.51 ± 0.08 nm/s. The velocity of the catalysts movement varied from 1.05 to 1.62 nm/s, much higher than that at 500 °C. Therefore, the increase of temperature enhanced both the oxidation rate of carbon and the mobility of the catalysts, that is, the catalysts became more active and more mobile at a higher temperature.

### Catalytic oxidation in 1 Pa dry air at 500 °C

**Figure 7:**
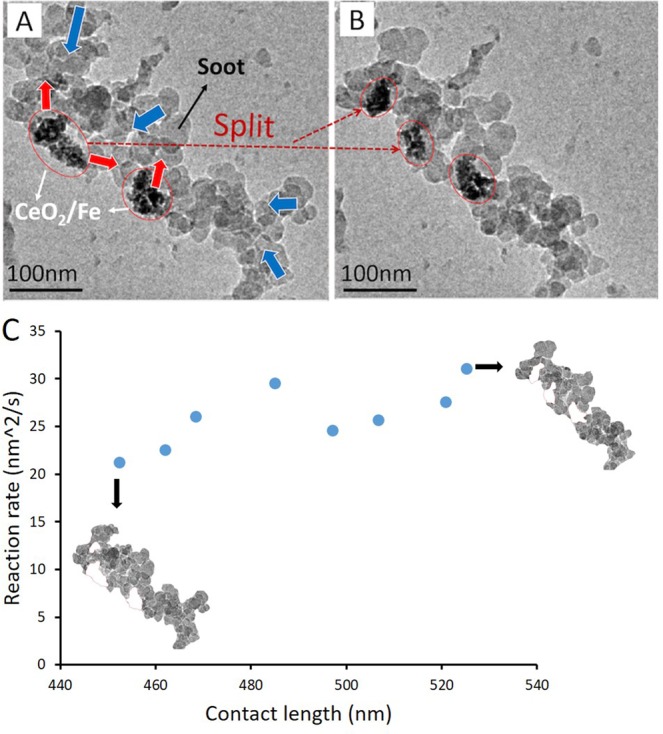
ETEM of carbon and 10%Fe/CeO_2_ during the reaction at 500 °C in 1 Pa air (~0.2 Pa O_2_); (**A**) 0 min; (**B**) 7 min. The 10%Fe/CeO_2_ catalyst clusters are circled in red lines. The movement of carbon and 10%Fe/CeO_2_ is marked by blue and red arrows, respectively. (**C**) Correlation of contact length and reaction rate ‘r’.

To investigate the effects of the partial pressure of oxygen on catalyst mobility and reaction, an *in-situ* ETEM experiment with 1 Pa dry air (~0.2 Pa O_2_, compared with 1 Pa pure O_2_ in section 3.2) at 500 °C was carried out. Two representative images are shown in Fig. 7(A,B). The observed phenomenon was similar as in previous two scenarios, involving the movement of the catalysts and carbon, and the split of the catalyst aggregate. During the reaction, the contact length between the carbon and catalyst varied from 452 to 525 nm and reaction rate ranged from 21.2 to 31.1 nm^2^/s. Figure 7(C) shows that with an increase of contact length between catalyst and carbon, the corresponding reaction rate ‘r’ also increases. The measured velocity of catalysts movement was in a range of 0.02–0.18 nm/s and the normalized reaction rate in 1 Pa dry air was calculated as 0.05 ± 0.01 nm/s. Both the velocity and the activity of the catalysts were lower than those at a higher temperature (800 °C) and in a higher O_2_ partial pressure (1 Pa), indicating that both temperature and oxygen partial pressure have influence on reaction rate and mobility.

### Mobility of catalyst nanoparticles and affecting factors

#### Dependence on the reaction rate

During the trials, we observed an interesting phenomenon which is the mobility of the 10%Fe/CeO_2_ catalysts towards carbon during the catalytic oxidation of this carbon. As shown in Fig. 3(E) and the supplementary video, the carbon particles were only consumed and oxidized at the carbon-catalyst interface, rather than on the entire outer surface of the carbon. Kamatani *et al*.^29^ have observed the reaction happened at the interface and found the mobile Ag species during carbon oxidation. The mobility of Ag is due to the liquefaction occurring at the Ag/SiO_2_ interface. However, in our study, the reaction temperature is far below the melting temperature of Ce and no visible reaction or movement of nanoparticles was observed in vacuum (Supplementary Information 7). This demonstrates that the catalyst nanoparticle movement is caused by the catalytic oxidation of carbon. In other words, the mobility of catalyst nanoparticles within this ETEM environment results from the oxidation of carbon, which subsequently re-constructs the carbon/catalyst interface to continue the reaction (possibly through minimizing the surface energy of the reactive interface).

**Figure 8:**
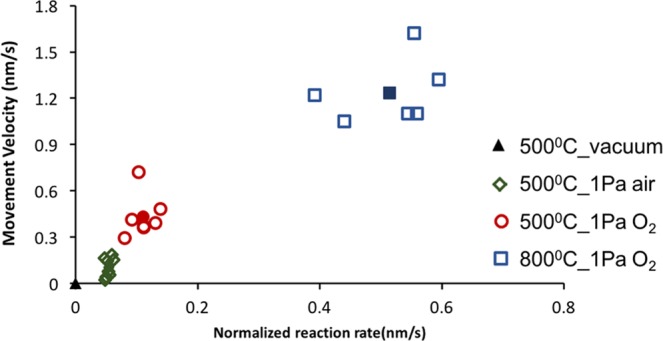
Correlation of the normalized reaction rate and the velocity of 10%Fe/CeO_2_ catalyst. The symbols with solid fill represent the average values.

The overview of the normalized reaction rate ‘r′’ and the catalyst movement velocity at different temperatures (500 °C and 800 °C) and for different O_2_ partial pressures is presented in Fig. 8 (with solid points representing the average value). Overall it suggests a positive relation between the normalized reaction rate and the mobility of catalyst; with a higher normalized reaction rate, the catalyst mobility is promoted. The normalized reaction rates and corresponding average movement velocities are shown in Table 1. The normalized reaction rate and catalyst mobility are sensitive to temperature and to a lesser extend O_2_ partial pressure. The comparison between reaction at 500 °C in 1 Pa O_2_ and air shows that a higher O_2_ partial pressure can increase the normalized reaction rate and hence the catalyst mobility. This is because the O_2_ partial pressure can affect oxygen adsorption on the ceria catalyst and diffusion to the reactive sites and thereby affect the oxidation rate^42^. The effect of temperature on catalytic carbon oxidation can be revealed by comparing the reaction in 1 Pa O_2_ at 500 °C and 800 °C. At a higher temperature, both the normalized reaction rate and catalyst mobility can be promoted. This is because an increase in temperature can facilitate the global oxidation process^27,43^. Since the normalized reaction rate is larger at a higher temperature, it is difficult to accurately measure the catalyst velocity. Considering the influence of these factors such as surface properties of the substrate, the nature and shape of the nanoparticles and the availability of carbon particles along the movement pathway, a largely fluctuating curve was obtained at the high temperature. Nevertheless, both O_2_ partial pressures and temperature were found to affect the reaction rate and catalyst mobility.

**Table 1:** Normalized reaction rates and corresponding catalyst velocities for different reaction conditions.

	Normalized rate (nm/s)	Movement velocity (nm/s)
500 °C, O_2_	0.11 ± 0.02	0.43 ± 0.14
800 °C, O_2_	0.51 ± 0.08	1.24 ± 0.21
500 °C, Air	0.05 ± 0.01	0.10 ± 0.06

In comparison of reaction with commercial CeO_2_ catalyst (Supplementary Information 4), where movement of catalyst was barely observed, our 10%Fe/CeO_2_ catalyst showed significant movement during the reaction, regardless of temperature and oxygen partial pressure. During the reaction with commercial CeO_2_ catalyst, the carbon particles showed movement towards the catalyst similar to a previous investigation^14^. However, only slight mobility of the catalyst was observed, emphasizing that only catalyst with certain properties can move during the reaction. Most importantly, this movement phenomenon could be attributed to the preferable normalized reaction rate of 10%Fe/CeO_2_ catalyst for carbon oxidation (avg. 0.12 nm/s), compared with normalized rate for CeO_2_ (avg. 0.024 nm/s). Since the mobility of catalyst results from rebuilding the carbon-catalyst interface in the process of carbon oxidation and is positively correlated with the normalized reaction rate, it is reasonable that the higher normalized reaction rate with 10%Fe/CeO_2_ catalyst leads to faster catalyst movement. Moreover, the spongy morphology of 10%Fe/CeO_2_ catalyst has a lower density or mass/size, which could have less friction with substrate and become easier to move. And this porous morphology could increase the contact points between carbon and catalyst and facilitate the oxygen diffusion to the catalyst/carbon interface, thus promoting reaction activity and catalyst movement^44^. Furthermore, adding Fe into ceria lattice could also enhance reaction rate due to good redox properties between Fe^3+^ and Fe^2+^ ^25^. With Fe incorporated into ceria, more oxygen vacancies on catalyst surface could be produced to keep the redox cycle continuing and provide more oxygen species for carbon oxidation. Figure 2(A) shows that catalyst particles of different sizes exist, including dispersed catalyst particles under 10 nm. Such dispersed nanoparticles increase the specific contact surface area between the catalyst and carbon particles and possibly increase the overall reaction rate. Therefore, another possible reason for the higher reaction rate for the Fe/CeO_2_ catalyst compared to that of the commercial CeO_2_ catalyst could be the presence of finely dispersed catalyst particles.

#### Driving forces

The movement of the catalyst and carbon nanoparticles can be explained by these forces among the nanoparticles including liquid bridging, electrostatic forces, thermophoresis, van der Waal forces and chemical bonding^45–47^. Liquid bridging is not considered as a driving force in reactions at 500 °C or 800 °C, as the mobility occurs during the high temperature reaction, which drives off any potential liquids. As for electrostatic or Coulombic forces; there is a known charging resulting from the high energy electron beam used by the TEM. This electron charging results in a negative charge in all particles, however, and repels the particles apart. Likewise, thermophoresis would also drive the particles apart - due to the exothermic oxidation reaction releasing hot products around the reaction zone, compared to the cold oxygen atmosphere elsewhere.

Despite all these repulsive or neutral forces, the catalysts and carbon move towards each other during the reaction, and actively react. Another possible source of electrostatic force is the transient states of catalysts during the catalysis. There are formations of oxygen vacancies, transformation of Ce^4+^/Ce^3+^ and Fe^3+^/Fe^2+^, and formation of O_2_^2−^/O^2−^ ^48^, probably resulting in local charge unbalance in the reaction interface of catalysts. But this electrostatic interaction was deemed too minimal to be the driving force, as the locally charged area is too small, the particle itself remains electroneutral, and no charged species were detected on carbon during the reaction in a previous study^17^. This leaves the possible driving forces as Van der Waals forces and chemical bonding, as they are related to the chemical nature of materials and always exist^45^.

#### DFT modeling on particle-particle interactions

**Figure 9:**
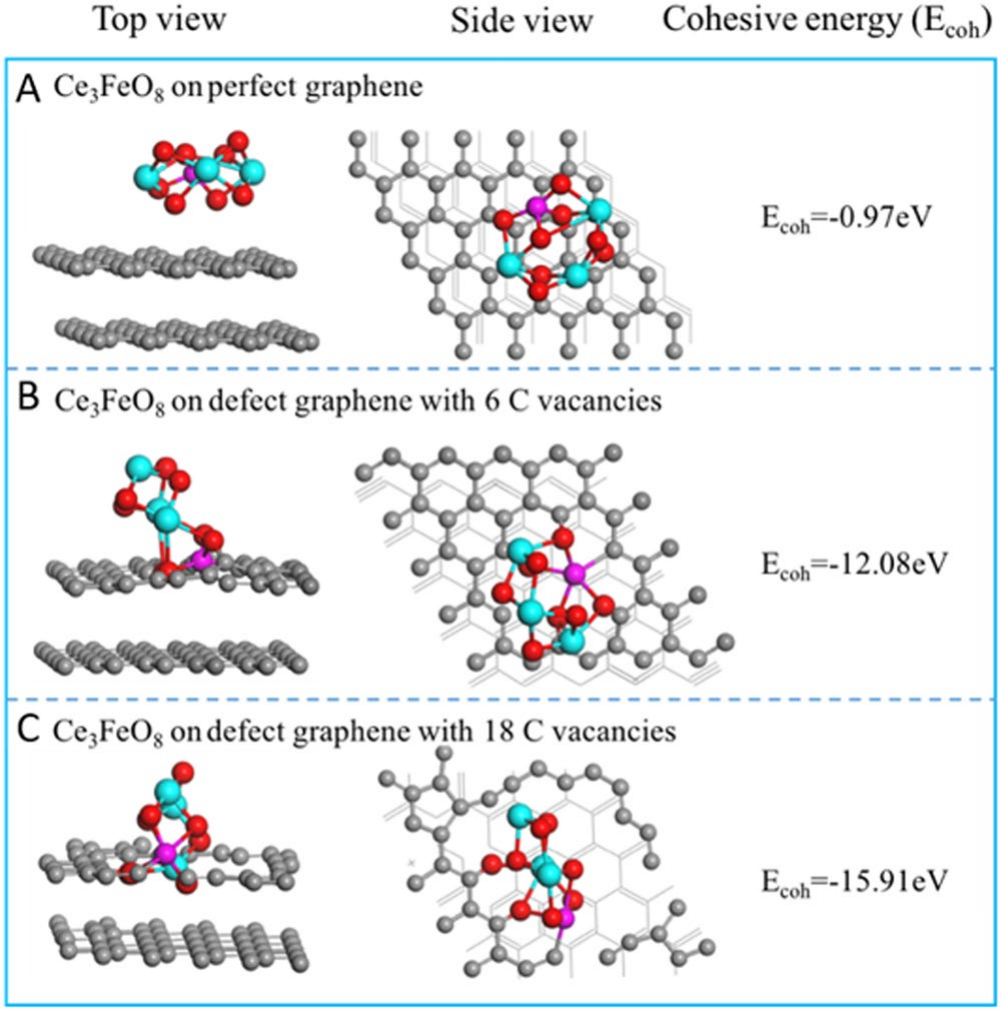
The most stable geometric structures (side and top views) of the adsorbed Ce_3_FeO_8_ cluster, and their corresponding cohesive energies (*E*_*coh*_, eV) on: (**A**) perfect; (**B**) 6-C defects and (**C**) 18-C defects graphene models. Cyan (Ce), red (O) and gray (C).

Chemical bonding is also considered to drive the movement of catalyst towards carbon particles. Figure 9 shows the DFT results from modeling the interactions between Fe/CeO_2_ and carbon particles. For Ce_3_FeO_8_ on perfect (or defect-free) graphene, no apparent bonding between the Ce_3_FeO_8_ cluster and surface C atoms was observed (Fig. 9A). It has a cohesive energy *E*_*coh*_ of −0.97 eV. However, when Ce_3_FeO_8_ interacts with the defect graphene edges, the O atoms in Ce_3_FeO_8_ forms covalent bonds with the defect C atoms (Fig. 9B,C). The cohesive energies *E*_*coh*_ are −12.08 eV and −15.91 eV for 6-C and 18-C defect graphene, respectively. These results suggest that the *in-situ* movement of the Fe/CeO_2_ nanoparticles might have resulted from the strong O-C bonding between CeO_2_ and the defective edge C in the carbon material during their continuous reaction.

## Conclusions

In this work, the performance of a 10%Fe/CeO_2_ catalyst towards carbon oxidation was investigated by observing *in-situ* the catalytic oxidation using ETEM. A semi-quantitative analysis was carried out to quantify the mobility of the particles and relate it to the catalyst activity. DFT calculations complemented this analysis to clarify the catalyst-carbon interactions. The main conclusions of this study are as follows:

- Both catalyst and carbon particles can be mobile. Typically, small catalyst agglomerates (50–100 nm) first move through nearly immobile carbon particles. As the amount of carbon decreases, these catalyst particles then slow done and eventually become immobile while mobile carbon particles move towards the catalyst. This relative mobility of the carbon and catalyst particles appears to be correlated to the size of those particles.
- Using 2D ETEM videos, reaction rates were calculated and found to be positively correlated to the contact length between carbon and catalyst particles, thus indicating that the catalytic carbon oxidation occurs at the carbon/catalyst interface.
- The normalized reaction rates and corresponding movement velocities of catalyst particles for reaction conditions of 500 °C in O_2_, 800 °C in O_2_, and 500 °C in air were found to be 0.11 ± 0.02 and 0.43 ± 0.14, 0.51 ± 0.08 and 1.24 ± 0.21, 0.05 ± 0.01 and 0.10 ± 0.06 nm/s, respectively. These data show a direct correlation between the normalized reaction rate and the movement velocity of catalyst (a larger normalized reaction rate would result in a greater movement velocity), suggesting that the motion of catalyst particles was caused by carbon oxidation on the catalyst’s surface. This data also confirms that the normalized rate and movement velocity of catalyst both increase with increasing the reaction temperature and oxygen partial pressure.
- DFT analysis revealed that there is a strong O-C bonding (12–16 eV) during the catalytic carbon oxidation process. This O-C bonding is likely an important contributor to the strong interactions between the Fe/CeO_2_ catalyst and carbon particles during the oxidation reactions, thereby driving the relative mobility of catalyst and carbon particles.

## Supplementary information

Real-Time Observation of Carbon Oxidation by Driven Motion of Catalytic Ceria Nanoparticles within Low Pressure Oxygen

1Pa O2 at 500C (our product)

1Pa O2 at 800C (our product)

1Pa dry air at 500C (our product)

1Pa O2 at 500C (commercial)
