# Supplementary material for: Real-Time Observation of Carbon Oxidation by Driven Motion of Catalytic Ceria Nanoparticles within Low Pressure Oxygen
Source: Sci Rep. 2019 May 30;9:8082. doi: 10.1038/s41598-019-44531-6 (PMC6542848; doi:10.1038/s41598-019-44531-6)
Supplement: Supplementary file 1 — Real-Time Observation of Carbon Oxidation by Driven Motion of Catalytic Ceria Nanoparticles within Low Pressure Oxygen [file 41598_2019_44531_MOESM1_ESM.docx]

Real-Time Observation of Carbon Oxidation by Driven Motion of Catalytic Ceria Nanoparticles within Low Pressure Oxygen

Boyu Li ^a, ‡^, Anton D. Sediako ^b, ‡^, Pei Zhao ^c^, Jingde Li ^a^, Eric Croiset ^a^, Murray J. Thomson ^b^, and John Z. Wen *^,a, c^

^a.^ Department of Chemical Engineering, University of Waterloo, 200 University Avenue West, Waterloo, ON, N2L 3G1, Canada

^b.^ Department of Mechanical & Industrial Engineering, University of Toronto, 5 King’s College Road, Toronto, ON, M5S 3G8, Canada

^c.^ Department of Mechanical & Mechatronics Engineering, University of Waterloo, 200 University Avenue West, Waterloo, ON, N2L 3G1, Canada

^‡^ contributed equally to this study.

* [john.wen@uwaterloo.ca](mailto:john.wen@uwaterloo.ca) phone: 1-519-888-4567 x38362

**1. Supplementary Videos**

1. Carbon and 10%Fe/CeO_2_ nanoparticles in the presence of 1 Pa O_2_ at 500°C – Video speed increased by 30 times
2. Carbon and 10%Fe/CeO_2_ nanoparticles in the presence of 1 Pa O_2_ at 800°C – Video speed increased by 30 times
3. Carbon and 10%Fe/CeO_2_ nanoparticles in the presence of 1 Pa dry air at 500°C – Video speed increased by 30 times
4. Carbon and commercial CeO_2_ nanoparticles in the presence of 1 Pa O_2_ at 500°C – Video speed increased by 30 times
5. **Catalyst manufacturing**

The catalyst was an iron doped cerium oxide compound, prepared by solution combustion synthesis (SCS) method. A stoichiometric solution of iron nitrate nonahydrate, cerium nitrate hexahydrate and glycine was stirred for 1 day under vigorous agitation to form the precursor, which was then combusted and calcined at 500^0^C for 5 h in air. This catalyst was labeled as 10%Fe/CeO_2_ with a specific surface area of 22.5 m^2^g^-1^. After grinding, the catalyst was particles with around 20 nanometers. Commercial CeO_2_ catalyst (Sigma-Aldrich, CAS:1306-38-3, particle size < 50 nm) were also used as a comparison.

1. **TGA experiments**

For comparison with the ETEM experiments, thermogravimetric analysis (TGA) experiments were conducted with a TA Instrument Q500 to study the reaction rate of the catalytic soot oxidation. Every TGA test follows the procedure below: 1) pre-treat 10 mg mixture at 150 ^0^C under inert gas for 30 min to remove water and other contaminants; 2) heat up the sample to the desired temperature (500, 800°C); 3) isothermal at the specific temperature for 20 min with air.

Reaction rate is calculated based on the equation below:

$$r=-\frac{dm}{dt}$$

where r is reaction rate, m is the remaining mass of the carbon black (mg) at time t (min).

The calculated reaction rate by using TGA at 500 ^o^C and 800 ^o^C were 0.0856 mg/min and 0.4438 mg/min. The ratio of rate at 800 ^o^C to rate at 500 ^o^C is 5.185. While the normalized reaction rate from ETEM at 500 ^o^C and 800 ^o^C were 0.12nm/s and 0.62nm/s, respectively, making the ratio become 5.167 (0.62/0.12). As 5.167 is similar to 5.185, the in-situ ETEM experiments are validated by the ex-situ TGA experiment.

1. **Catalytic oxidation in 1 Pa O2 at 500°C with commercial CeO_2_ catalyst**

A similar ETEM experiment was done with mixture of commercial CeO_2_ catalyst and carbon in 1 Pa O_2_ at 500°C, as shown in Figure S1. The movement of catalyst can be barely observed, which is different from the scenario with our 10%Fe/CeO_2_ catalyst. The contact length between carbon and catalyst ranged from 629-822 nm, and the measured normalized reaction rate is in a range of 0.015-0.03 nm/s with an average of 0.024 nm/s, suggesting a lower reaction rate than the 10%Fe/CeO_2_ catalyst at the same reaction conditions. This low oxidation rate could be the reason that catalyst movement is not observed.


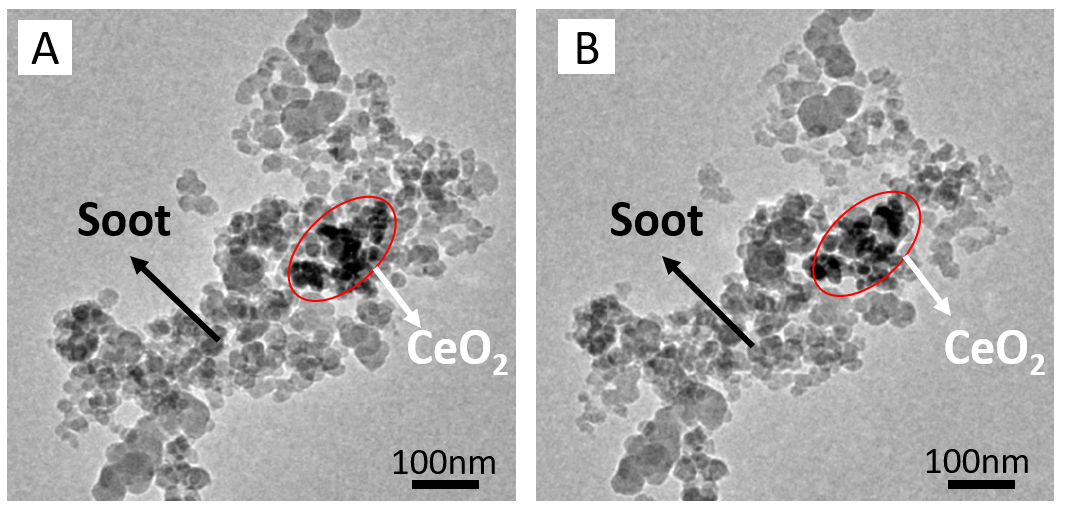


Figure S1 ETEM of carbon and commercial CeO2 during the reaction at 500℃ in 1Pa O2; A: 0min; B: 24min. The CeO2 catalyst clusters are circled in red lines.

1. **The normalized reaction rate as a function of time**

The appropriateness of the normalized rate calculation as a function of time is shown in Figure S2, showing that the normalized rate remains reasonably constant during the initial period of the reaction. This Figure also helped identifying a few outliers that were removed for subsequent calculations.

**
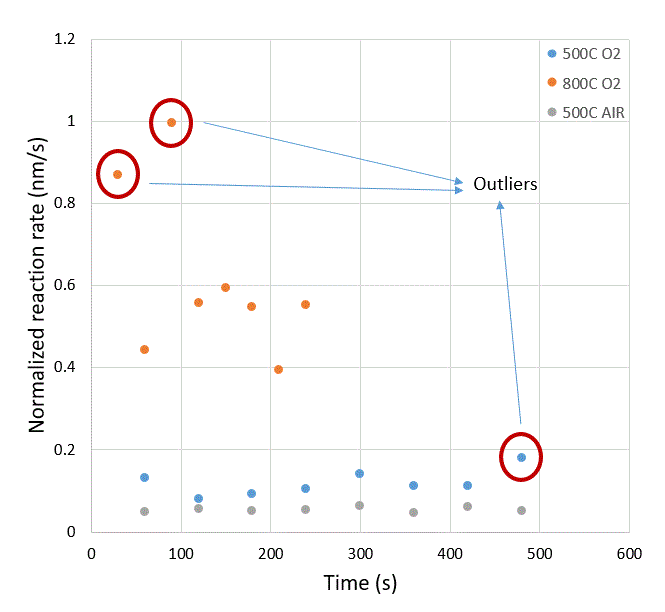
**

Figure S2. The normalized reaction as a function of time.

1. **Sensitivity analysis**

Due to the sensitive nature of nanoparticles, where conventionally negligible forces can play an enormous role, a sensitivity analysis must be done for such work to establish that the effects observed are not due to the effect of the electron beam or gas flows.

- 1. **Beam Effects**

Although the electron beam within ETEM would effect the carbon oxidation, but in order to establish that the observed movement is not the result of beam charging, a sample of carbon and catalyst nanoparticle mixture was heated up to 500°C in high vacuum. Images were taken, the beam was turned off, and oxygen injection started. Figure S3(A) was recorded just before turning off the beam. After 40 minutes reaction, the beam was turned on again and image was recorded as shown Figure S3 (C). Since Figure S3 (A) and (C) were not able to clearly show the catalyst configuration, Figure S3 (B) and (D) - the dark field image corresponding to Figure S3 (A) and (C) respectively, were used to exhibit the movement of catalyst. It can be observed that carbon originally in contact with the catalyst was consumed by the time Figure S3 (C) was taken, and the catalyst circled in red has obviously moved significantly and assembled into a larger aggregate. This demonstrated that the movement of the catalyst is not the result of beam energy, but rather by the reactions between the catalyst and carbon nanoparticles.


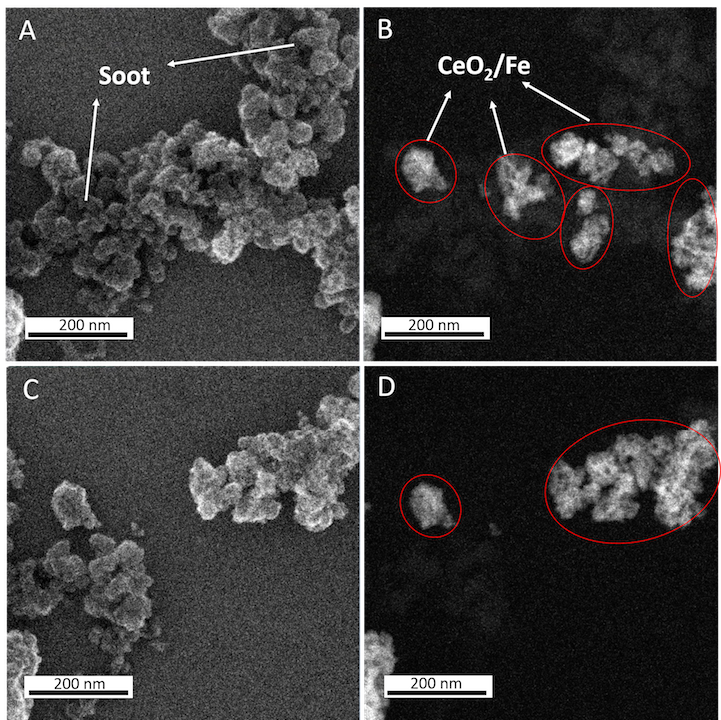


Figure S3 ETEM images for carbon and CeO2/Fe catalyst nanoparticles recorded at 500 ℃ in 1 Pa O2, A and B: Secondary electron image and corresponding dark field image before turning off beam, respectively; C and D: Secondary electron image and corresponding dark field image after 40 min

- 1. **Gas Flow and Substrate Effects**

As the sample is in a constant cloud of replenishing reactant gas, it is necessary to demonstrate that the mobility of catalyst was not caused by gas flow. Initial calculations and experiments have established that the sample is well clear of the mean free path of the gas, and can be considered to sit in a quiescent atmosphere of gas [S1]. These trials were repeated with a catalyst/carbon sample at 500°C and 1 Pa of N_­­­2_ flow, as shown in Figure S4. There was no obvious change or movement of catalyst and carbon, which meant that no carbon oxidation or catalyst movement happened in the N_2_ atmosphere. It confirmed that the carbon oxidation and movement of catalyst were not caused by gas flow, nor any potential reactions with the substrate.


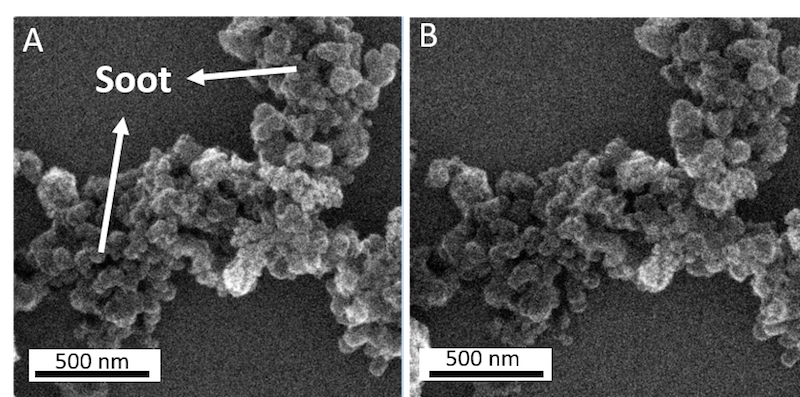


Figure S4: ETEM images for carbon and SCS catalyst nanoparticles recorded at 500 ℃ in 1 Pa N2, A: 0 min, B: 60 min

- 1. Secondary Reactions and Movement

In order to further understand catalyst mobility, it was necessary to justify if the movement of the catalyst was caused by the reaction between the particles themselves. To check this influence, tests were run with a sample of the catalyst and carbon nanoparticles separately at 500$℃$ in 1 Pa oxygen atmosphere. As shown in Figure S5, for catalyst only, no obvious change of the catalyst was observed after 25 minutes. In Figure S6, non-catalytic carbon oxidation only happened from the outer surface of carbon nanoparticles and no movement was observed during 40 minutes reaction. This shows that only with catalytic carbon oxidation, both carbon and catalyst nanoparticles would show mobility.


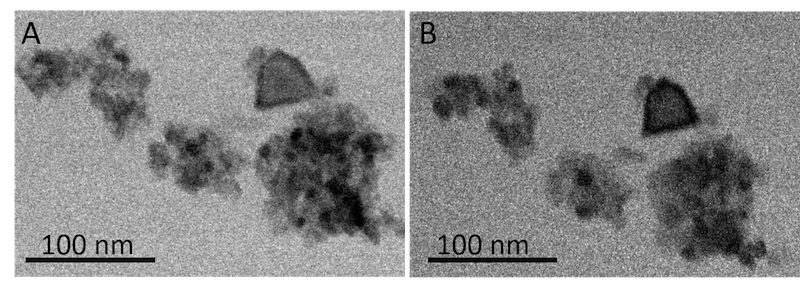


Figure S5: ETEM images for CeO2/Fe catalyst only recorded at 500 ℃ in 1 Pa O2, A: 0 min, B: 25 min


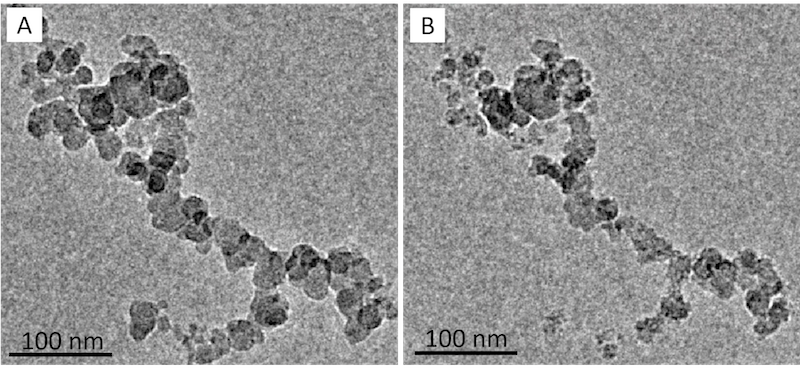


Figure S6: ETEM images for non-catalytic carbon oxidation recorded at 500 ℃ in 1Pa O2, A: 0 min, B: 40 min

- 1. **In high vacuum**

Finally, a study was done to establish the reactivity of the catalyst with no oxygen injection. For the reactions in vacuum, as recorded in Figure S7, the carbon-catalyst mixture was heated up to 500°C and maintained at this temperature for 10 minutes at high vacuum. Without oxygenated atmosphere, extremely slow oxidation of carbon, as well as the movement of carbon and catalyst nanoparticles, could be observed because the catalyst itself can provide oxygen to carbon.


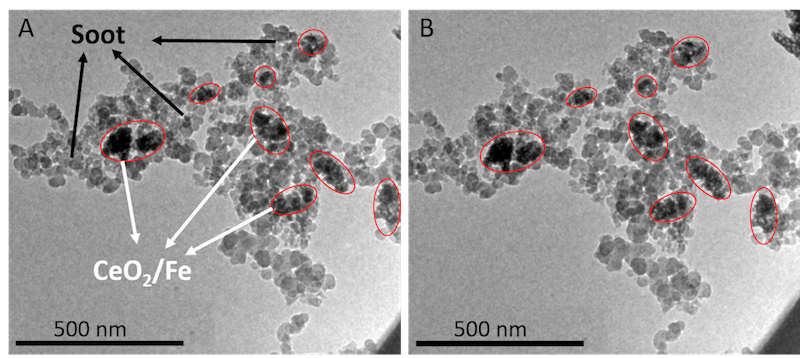


Figure S7: ETEM images for carbon and catalyst nanoparticle mixture recorded at 500℃ in vacuum: A: 0 min, B: 10 min

1. **Uncertainty analysis:**
2. Distinguishing background and carbon particles are somewhat difficult because they are all in grey color.
3. Distinguishing boundary of catalyst may induce error since the boundary are determined by human eyes.
4. Since 3D image was converted to 2D image, we assume there are only one layer of carbon, but in some area there are possibly not only one layer of carbon.
5. When calculating the reaction rate at the interface, the contact length may change during a time period. But the contact length we used to calculate the reaction rater is the length at the beginning or end of that time period.

**References**

[S1] Sediako, A.D., et al., *Real-time observation of soot aggregate oxidation in an Environmental Transmission Electron Microscope.* Proceedings of the Combustion Institute, 2016.
